# MVP-Associated Filamin A Mutations Affect FlnA-PTPN12 (PTP-PEST) Interactions

**DOI:** 10.3390/jcdd2030233

**Published:** 2015-09-08

**Authors:** Damien Duval, Pauline Labbé, Léa Bureau, Thierry Le Tourneau, Russell A. Norris, Roger R. Markwald, Robert Levine, Jean-Jacques Schott, Jean Mérot

**Affiliations:** 1 Institut du thorax, Inserm UMR 1087, CNRS UMR 6291, 8 Quai Moncousu, Nantes F-44007, France; E-Mails: damien.duval@etu.univ-nantes.fr (D.D.); pauline.labbe@etu.univ-nantes.fr (P.L.); bureau.lea@gmail.com (L.B.); thletourneau@yahoo.fr (T.L.T.); jjschott@univ-nantes.fr (J.-J.S.); 2 Department of Cell Biology and Anatomy, Medical University of South Carolina, Charleston, SC 29425, USA; E-Mails: norrisra@musc.edu (R.A.N.); markwald@musc.edu (R.R.M.); 3 Harvard Medical School, Massachusetts General Hospital, Boston, MA 02114-2696 USA; E-Mail: rlevine@partners.org

**Keywords:** Filamin-A, PTPN12, mitral valve prolapse, p190RhoGAP, Src

## Abstract

Although the genetic basis of mitral valve prolapse (MVP) has now been clearly established, the molecular and cellular mechanisms involved in the pathological processes associated to a specific mutation often remain to be determined. The *FLNA* gene (encoding Filamin A; FlnA) was the first gene associated to non-syndromic X-linked myxomatous valvular dystrophy, but the impacts of the mutations on its function remain un-elucidated. Here, using the first repeats (1–8) of FlnA as a bait in a yeast two-hybrid screen, we identified the tyrosine phosphatase PTPN12 (PTP-PEST) as a specific binding partner of this region of FlnA protein. In addition, using yeast two-hybrid trap assay pull down and co-immunoprecipitation experiments, we showed that the MVP-associated FlnA mutations (G288R, P637Q, H743P) abolished FlnA/PTPN12 interactions. PTPN12 is a key regulator of signaling pathways involved in cell-extracellular matrix (ECM) crosstalk, cellular responses to mechanical stress that involve integrins, focal adhesion transduction pathways, and actin cytoskeleton dynamics. Interestingly, we showed that the FlnA mutations impair the activation status of two PTPN12 substrates, the focal adhesion associated kinase Src, and the RhoA specific activating protein p190RhoGAP. Together, these data point to PTPN12/FlnA interaction and its weakening by FlnA mutations as a mechanism potentially involved in the physiopathology of FlnA-associated MVP.

## 1. Introduction 

The mitral valve prolapse (MVP), the most common form of cardiac disease, involves approximately 2%–3% of the population and remains one of the major causes of valve surgery. In parallel to syndromic forms, the familial inheritance of non-syndromic MVP has now been clearly established [1]. Indeed, the syndromic forms of MVP have been linked to several genes such as *FBN1* (Fibrillin-1) in Marfan syndrome [2], *TGFBRs* (TGFβ receptors) in Loeys-Dietz syndrome [3], *COL-1* (Collagen-1) in Ehlers-Danlos syndrome [4], while the *FLNA* (Filamin A) gene was associated to non-syndromic X-linked myxomatous valvular dystrophy (XMVD) [5,6]. However, although recent studies have unraveled the molecular, cellular, and physiopathological processes in few syndromic MVPs, including Marfan syndrome, and lead to potential therapeutic treatments for better healthcare of these patients [7,8], the deleterious mechanisms at work in FlnA-associated MVP remain to be elucidated.

In the late 1990s we mapped X-linked myxomatous valvular dystrophy to chromosome Xq28 gene locus in a large French family and identified in 2007, in all affected members, a first mutation encoding p.Pro637Gln residue substitution (P637Q) in the *FLNA* gene [5,6]. In addition, three other FlnA mutations encoding two residue substitutions, p.Gly288Arg (G288R), p.Val711Asp (V711D), and a 1944-bp genomic deletion were subsequently identified in four other families around the world. Although FlnA mutations have been associated to important and various congenital developmental diseases, including periventricular heterotopy (PVH) [9], Melnick-Needles syndrome (MNS) [10], and otopalatodigital syndrome (OPD), it is important to note that the patients bearing MVP-associated FlnA mutations only suffer from valvular affections, suggesting similar but specific mechanisms are at work for these MVP-associated FlnA-mutations. As a matter of fact, common clinical traits were shared in the different families, including early onset of poly-valvular defects sometimes detectable in newborns, typical features of myxomatous disease with marked thickening of the mitral valve (thickness was superior to 4 mm), and alterations of the sub-valvular apparatus. Interestingly, we also detected similar defects in targeted FlnA knockout mice model which exhibited abnormal valvular development and hyperplasic mitral valves, strongly suggesting that the MVP FlnA mutations we identified are loss of function mutations [11,12].

Filamin A (FlnA) is the first actin filament cross-linking protein identified in non-muscle cells. It organizes actin filaments in orthogonal networks to stabilize the cellular actin cortex, and many previous studies defined central roles for FlnA in mechano-protection, cell adhesion, spreading, and migration [13,14,15,16,17]. The FlnA protein consists of a conserved N-terminal actin binding region followed by 24 immunoglobulin-like (Igl) repeated domains, among which the 24th is involved in non-covalent protein dimerization [15]. Over 90 FlnA interacting protein partners have now been identified attesting to the implication of FlnA in the regulation of many signaling cascades involving actin cytoskeleton regulation. Interestingly, only few of these binding partners were shown to interact with the first N-terminal Igl repeats region of FlnA (Igl-1–8) that are targeted by the MVP-associated FlnA mutations suggesting the pathological effects of the latter may arise from interactions with new, yet unknown, binding partners [18,19].

**Figure 1:**
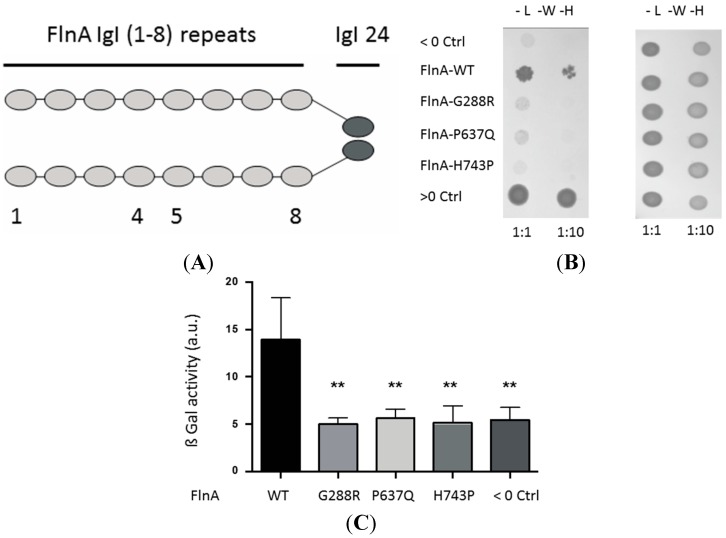
Effects of Filamin A mutations on FlnA/PTPN12 interaction. Yeast two-hybrid assay. (**A**) Schematic cartoon of the bait used in the two-hybrid library screening and interaction assay of FlnA mutations. The bait consists in the first eight Igl repeats fused to the second hinge and the 24th repeat of FlnA; (**B**) the left-hand image shows yeasts co-transfected with *GAL4*-AD-PTPN12 and, from top to bottom lane, *GAL4*-BD-CT7 (negative control; Neg Ctrl), *GAL4*-BD-FlnA-WT, G288R, P637Q or H743P or co-transfected with *GAL4*-AD-PICK1 and *GAL4*-BD-CT7 (positive control, Pos Ctrl) growing on histidine-free medium (interaction assay) or histidine-supplemented plates (growth assay, right-hand image). Two dilutions of 1:1 and 1:10 were spotted; (**C**) quantification of FlnA/PTPN12 interaction in β galactosidase assay. Results are presented in arbitrary units (au). ** indicates significant differences (*p* < 0.01, *n* = 4) *vs.* FlnA-WT.

To identify binding partners specifically interacting with the FlnA region targeted by the MVP mutations we performed a yeast two-hybrid screen of a cDNA library using the FlnA Igl repeats 1–8 region as a bait (Figure 1A). This screen identified a key regulator of cellular cytoskeleton and cell-extracellular matrix interaction signaling pathway, the tyrosine phosphatase non-receptor type PTPN12/PTP-PEST, as a binding partner of FlnA-1–8 repeats [20,21,22]. Furthermore, using yeast two-hybrid, pulldown and co-immunoprecipitation assays, we showed that FlnA-G288R and P637Q mutations and a recently-identified mutation p.His743Pro (H743P) affect FlnA/PTPN12 interactions, supporting a potential role of altered FlnA/PTPN12 interactions in the physio-pathological mechanisms of FlnA-associated MVP.

## 2. Experimental Section

### 2.1. cDNAs, Mutagenesis, and Reagents

The cDNA encoding FlnA Igl repeats 1–8 fused to its Hinge 2 domain and its 24th Igl repeat (FlnA-1–8,24, see Figure 1A), was a kind gift of Dr F. Nakamura [15]. Human PTPN12 cDNA was obtained from Source Bioscience (Nottingham, UK) and C-terminally EGFP tagged FlnA from Dr D. Calderwood (Yale University, New Haven, CT, USA). pPC97, pPC86 vectors and the pPC97-CT7 (encoding the *GAL4* DNA binding domain fused to the C-terminal tail of mGluR7a glutamate receptor *GAL4*-BD-CT7), and pPC86-PICK1 (encoding *GAL4*-AD-PICK1 fusion) constructs used as positive controls in interaction assay were a kind gift of Dr H. Boudin (INSERM U913, Nantes, France) [23]. The construct encoding GST-RhoQ63L was a kind gift of Dr C. Guilluy (INSERM U1087, Nantes, France). All the mutations described below were made using PCR-based mutagenesis kits (Quickchange kit from Agilent Tech, Les Ulis, France). Polyclonal antibodies for GAPDH (1:10,000) and HRP-conjugated antibodies (1:10,000) were purchased from Santa Cruz. Monoclonal antibodies were purchased from Chemicon (anti-filamin A, 1:1000, Merck Millipore, Milsheom, France), Clontech (anti-EGFP, 1:1000, Saint-Germain-en-Laye, France), Roche (anti-HA, 1:1000), BD Bioscience (p190RhoGAP, 1:500, Le Pont de Claix, France), and Cell Signaling (Src and phospho-Src-Y416, 1:500 both, Merck Millipore).

### 2.2. Cell Culture and Transfection

The maintenance and the production of melanoma cells stably transfected with wild-type and mutant FlnAs were previously described in [24]. Hek293 cells were cultured in DMEM, supplemented with 10% fetal calf serum (FCS) and l-glutamine. The cells were transfected using Lipofectamine (Invitrogen, Illkirch, France) or Genecellin (BiocellChallenge, Toulon, France) according to the manufacturers’ specifications.

### 2.3. Migration Assays: Wound Healing Assays

In wound healing assays, confluent cell monolayers were scratched with a pipette tip, washed with phosphate-buffered saline (PBS), and cell migration monitored using a Leica DMI6000B microscope (Nanterre, France) equipped for time-lapse video microscopy. Wound closure was monitored for 24–28 h on three fields per well and the data are presented as % of closure of FlnA-WT cells’ monolayer wound closure at *t* = 24 h. Snapshots of migrating cells presenting trailing tails were extracted from these experiments.

### 2.4. Yeast Two-Hybrid Library Screening

Yeast two-hybrid screening was performed by Hybrigenics (Hybrigenics Services, Paris, France). The coding sequence of FlnA-1–8,24 was PCR-amplified and cloned into pB27 as a C-terminal fusion to LexA (*N*-LexA-FlnA-1–8,24-C). The construct was checked by sequencing the entire insert and used as a bait to screen a random-primed human placenta cDNA library constructed into pP6. pB27 and pP6 derive from the original pBTM116 and pGADGH plasmids, respectively. 79.4 million clones (eight-fold the complexity of the library) were screened using a mating approach with YHGX13 (Y187 ade2-101:loxP-kanMX-loxP, matα) and L40ΔGal4 (matα) yeast strains as previously described in [25]. 129 His + colonies were selected on a medium lacking tryptophan, leucine, and histidine. The prey fragments of the positive clones were amplified by PCR and sequenced at their 5′ and 3′ junctions. The resulting sequences were used to identify the corresponding interacting proteins in the GenBank database (NCBI, http://www.ncbi.nlm.nih.gov/) using a fully automated procedure. A confidence score (PBS, for Predicted Biological Score) was attributed to each interaction as previously described in [26]. The PBS relies on two different levels of analysis. Firstly, a local score takes into account the redundancy and independency of prey fragments, as well as the distribution of reading frames and stop codons in overlapping fragments. Secondly, a global score which takes into account the interactions found in all of the screens performed at Hybrigenics using the same library. This global score represents the probability of an interaction being nonspecific.

### 2.5. Yeast Two-Hybrid Interaction Assays

WT-FlnA-1–8,24 cDNA and those encoding G288R, H743P, and P637Q mutations (G288R, H743P, P637Q-FlnA-1–8,24, respectively) were PCR amplified and cloned in frame (5′-SalI, NotI-3′) with the DNA binding domain of *GAL4* in the pPC97 derived vector containing *LEU2* selection reporter (*GAL4*-BD-FlnA). The cDNA encoding the T587-N753 peptide region of PTPN12 identified in the library screen was PCR amplified and cloned in frame (5′-SalI, NotI-3′) with the activation domain of *GAL4* in the pPC86 vector containing *TRP1* selection reporter (*GAL4*-AD-PTPN12). The yeast reporter strain PJ69-4A was used and sequentially transfected with recombinant pPC97 and pPC86 plasmids using the lithium acetate method. Cells were patched onto selective (leucine; tryptophan and histidine-deprived L^−^W^−^H^−^) plates that were incubated at 30 °C for 2–6 days. Standard colorimetric β-galactosidase assays performed on liquid cultures were used to verify and quantify the strength of the interaction between the different binding partners tested.

### 2.6. Co-Immunoprecipitation and Immunoblotting

Hek293 cells transfected with C-terminally EGFP-tagged FlnA-WT or FlnA-P637Q were lysed in NETF buffer containing: 100 mM NaCl, 2 mM EGTA, 50 mM Tris pH 7.5, 50 mM NaF, 1% NP-40, 1 mM PMSF, 1 mM Na_3_VO_4_, 1× protease inhibitor cocktail (Roche, Boulogne-Billancourt, France), and lysates clarified by centrifugation (15,000× *g* for 15 min at 4 °C). The cell lysates (500 µg) were incubated with 6 µg of anti-HA for 2 h at 4 °C and then with 30 µL of protein A conjugated beads (Dynabeads, Invitrogen, Illkirch, France) for 1 h at 4 °C. The immunoprecipitates were washed four times with NETF buffer, separated by SDS-PAGE, and transferred to a nitrocellulose membrane (Bio-Rad Transblot, Marnes-la-Coquette, France). Immunoblots were probed with appropriate antibodies and revealed using an enhanced chemiluminescence kit (GE Healthcare, Buc, France). Chemiluminescence signals were quantified using an Imager system (Roche Diagnostic, Boulogne-Billancourt, France) and the data normalized with respect to GAPDH. 

### 2.7. Pulldown Experiments

The cDNA fragment encoding the peptide region of hum-PTPN12 (Pro600-Asp709) was PCR amplified and cloned in PGEX-6P1 vector to produce GST-PTPN12 fusion protein. The GST-PTPN12 and GST-PTPN12-ΔPro in which the proline-rich region (674-PPPLPERTP-682) of PTPN12 was deleted, were produced in BL21 *Escherichia coli* (*E. Coli*) and purified using glutathione agarose 4B beads (Macherey-Nagel, Hoerdt, France) as previously described in [24]. The GST-fusion proteins (30 µg) were incubated for 1 h at 4 °C with 500 µg of cleared cellular lysates prepared from melanoma cells stably transfected with FlnA-WT or FlnA-P637Q in NETF buffer containing: 100 mM NaCl, 2 mM EGTA, 50 mM Tris pH 7.5, 50 mM NaF, 1% NP-40, 1 mM PMSF, 1 mM Na_3_VO_4_, and 1× protease inhibitor cocktail (Roche). The beads were washed four times with cell lysis buffer and bound proteins separated by SDS-PAGE. Bound FlnAs were detected by immunoblotting using an anti-FlnA antibody. A similar protocol using GST-RhoA-Q63L and anti-p190RhoGAP antibody was performed to test p190RhoGAP activation status. 

## 3. Results

### 3.1. A Yeast Two-Hybrid Library Screen Identified PTPN12 as A FlnA Binding Partner

Based on (1) the observation that all MVP-associated FlnA mutations target the same N-terminal region of the protein namely: the Igl-1 (G288R), Igl-4 (P637Q), Igl-5 (V711D and H743P), and Igl-6–7 (Δ761-943); (2) the fact that FlnA-associated MVP patients shared very similar clinical features; (3) the notion that the dimerization of FlnA polypeptides determines its association with specific binding partners [27]; and (4) the impossibility to use the full-length FlnA because of its size, we chose to use the first eight repeats of FlnA fused to its dimerization domain (24th Igl) as a bait to screen a human placental cDNA library in a LexA-based yeast two-hybrid assay (Figure 1A). 79.4 million clones (eight-fold the complexity of the library) were screened and HIS-positive clones isolated on leucine-, tryptophan-, and histidine-free selection medium. The sequencing of one of the positive clones identified the region (residue 587–753) of the tyrosine phosphatase PTPN12/PTP-PEST. Interestingly, this domain of PTPN12 encodes a proline-rich motif that was shown to participate to FlnA/PTPN12 interaction, suggesting the Igl repeat 1–8 region of FlnA constitute its yet unknown interaction domain with PTPN12 [28]. We then tested the impact of the FlnA-MVP mutations on FlnA/PTPN12 interactions.

### 3.2. MVP-Associated FlnA Mutations Impede its Interactions with PTPN12

#### 3.2.1. Yeast Two-Hybrid Assay

To confirm the FlnA/PTPN12 interaction we observed using the LexA-based two-hybrid system, and to test the effects of the mutations on this interaction, we used another yeast two-hybrid assay based on the *GAL4* transcription factor. As illustrated on Figure 1, the yeasts co-expressing *GAL4*-AD-PTPN12 and *GAL4*-BD-FlnA-WT grew on the selective L^−^W^−^H^−^ plates (Figure 1B, second lane) although less rapidly than those co-transfected with the two strongly-interacting partners used as a positive control, *GAL4*-AD-Pick1 and *GAL4*-BD-CT7 (Figure 1B, bottom lane). These data confirmed the direct PTN12/FlnA interaction and showed it could also be detected using this interaction trap assay. We then tested the effects of the MVP-FlnA mutations. Interestingly, neither FlnA-G288R, P637Q, H743P, nor CT7 (used as a negative control, top lane) enabled growth of PTPN12-expressing yeasts in the L^−^W^−^H^−^ selection medium (Figure 1B). Importantly, all the yeasts grew equally well when the medium was supplemented with histidine, excluding potential toxicity biases due to exogenous protein overexpression (Figure 1B, right-hand image). The loss of interaction was quantified using β-galactosidase assay performed on liquid culture. As shown on Figure 1C, the FlnA mutations leveled down the galactosidase activity, which represents the PTPN12/FlnA-WT binding strength, to that measured with the negative control (CT7), suggesting a complete loss of interaction with PTPN12.

#### 3.2.2. Pulldown and Co-Immunoprecipitation Assays

**Figure 2:**
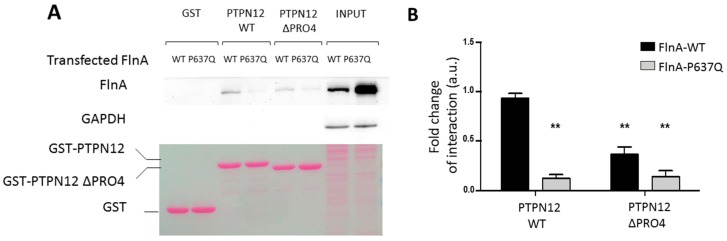
Effects of Filamin A mutations on FlnA/PTPN12 interaction. A) Pull-down assay. Equal amounts of GST, GST-PTPN12 or GST-PTPN12-ΔPro (lower image Red Ponceau coloration) were incubated with lysates of cells expressing FlnA-WT or FlnA-P637Q. Despite much larger expression of P637Q mutant in this experiment (input lanes), only FlnA-WT was readily detectable in GST-PTPN12 pulldown (lanes three and five). Deletion of the proline-rich domain of PTPN12 abolished PTPN12/FlnA interaction. Note the absence of detectable GAPDH contamination in the pulldown assays, which attests to low background and absence of unspecific binding in these experiments. B) Quantification from four similar experiments. ** indicates significant differences in the amount of retained FlnAs *vs.* FlnA-WT using GST-PTPN12 (*p* < 0.01, *n* = 4).

FlnA/PTPN12 interactions were then challenged in a pulldown assay using the interaction domain of PTPN12 fused to GST protein and melanoma cell lines stably expressing FlnA-WT and FlnA-P637Q [24]. In this assay (Figure 2), FlnA-WT was specifically retained on GST-PTPN12 (Figure 2, lane three) but not by GST or the mutant GST-PTPN12 construct in which the proline-rich domain (674-PPPLPERTP-682) of PTPN12 previously shown to participate to PTPN12-FlnA interactions (GST-PTPN12-ΔPro), was deleted (Figure 2 lanes one and five, respectively). Importantly, FlnA-P637Q did not interact with any of these GST constructs (Figure 2) even when larger amount of FlnA-P637Q was loaded on the beads (see input signals). Quantification revealed over 75% reduction of the FlnA-P637Q pulled down with respect to FlnA-WT (histogram on Figure 2).

**Figure 3:**
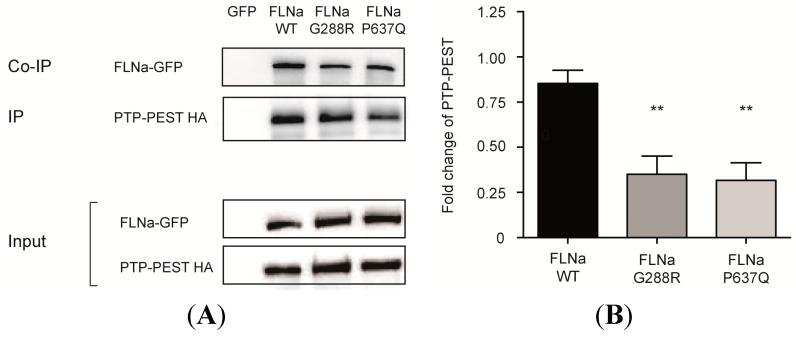
Effects of Filamin A mutations on FlnA/PTPN12. (**A**) Co-immunoprecipitation assay. HA-tagged PTPN12 (HA-PTPN12) was co-transfected with GFP or GFP-tagged-FlnAs in Hek293 cells and immunoprecipitated using anti-HA antibody (IP). G288R and P637Q mutations significantly reduced the amount of FlnA co-IPed with PTPN12 (Co-IP) (lanes 3,4 upper blot). (**B**) Quantification of four similar experiments. ** indicates significant differences in the amount of co-immunoprecipitated FlnAs *vs.* FlnA-WT (*p* < 0.01, *n* = 4).

The interactions were then tested in a cellular context. Full-length HA-tagged PTPN12 was co-transfected with GFP-tagged-FlnA-WT, G288R or P637Q in Hek293 cells. As shown in Figure 3, both mutations significantly reduced by more than 60% (*p* < 0.01, *n* = 4) the FlnA co-immunoprecipitated with HA-PTPN12 using anti-HA antibody.

Together, these data are consistent with a model according to which FlnA repeats domain 1–8 constitute a structural element that interacts with the proline-rich region of PTPN12, and that the mutations G288R, P637Q, and potentially H743P, affect this molecular interface. We then analyzed the effects of FlnA mutations on the phosphorylation status of PTPN12 target substrates potentially affected in the physiopathological context of MVP.

### 3.3. PTPN12-FlnA Interactions Participate to p190RhoGAP and Src Regulation

**Figure 4:**
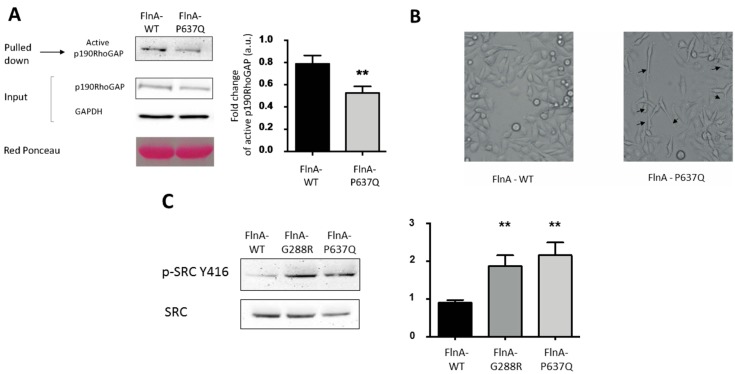
FlnA mutations increase p190RhoGAP activation and Src phosphorylation. (**A**) Active RhoA activating proteins were pulled down using GST-RhoA-Q63L constructs from melanoma cells expressing FlnA-WT or P637Q and the amount of p190RhoGAP retained, quantified by Western blotting. The upper blot shows less p190RhoGAP was retained from FlnA-P637Q cells. ** indicates significant differences in the amount of retained p190RhoGAP in FlnA-P637Q *vs.* FlnA-WT cells (*p* < 0.01, *n* = 4); (**B**) phase contrast images of migrating FlnA-WT and P637Q transfected melanoma cells. Note the long and persistent trailing tails observed in FlnA-P637Q cells (arrow heads); and (**C**) phosphorylation of tyrosine Y416 of Src (phospho-Src-Y416) was significantly increased in FlnA-G288R and P637Q transfected cells *vs.* FlnA-WT cells. ** indicates significant differences *vs.* FlnA-WT (*p* < 0.01, *n* = 4).

In our previous studies we showed that the MVP-FlnA mutations tilt the RhoA/Rac1 GTPases balance toward increased RhoA activity and impede the cellular adhesion and migration capacities [24]. On the other hand, PTPN12 is known to regulate the tyrosine phosphorylation status and the activity of key actors of the RhoA GTPase pathway, extracellular matrix/cell interaction, cell adhesion, and migration signaling pathways, including the RhoA GTPase activating protein p190RhoGAP and the Src family kinase [20,29]. We, thus, investigated the impact of the FlnA mutations on these two actors. p190RhoGAP activity was tested in a set of pulldown experiments using the GST fused to activated RhoA as a bait (GST-RhoA-Q63L) to specifically isolate active RhoA-GAP regulators, including p190RhoGAP [30]. As shown in Figure 4A, and consistent with an increased cellular RhoA activity [24], a significantly lower amount of p190RhoGAP (40% decrease *p* < 0.01, *n* = 4) was isolated in FlnA-P637Q cells compared to FlnA-WT cells. On the other hand, the close inspection of cell morphologies during migration corroborated these results and suggested that FlnA-PTPN12 interaction might participate to PTPN12 targeting to specific subcellular domains. Indeed, as shown in Figure 4B, P637Q (and FlnA-G288R not shown) cells exhibited persistent and long trailing tails compared to FlnA-WT cells. Puzzling enough, this feature was reminiscent of the observation made by Sastry and colleagues according to which PTPN12 knock down fibroblasts also “develop long, thin tails in the rear” [29]. One can speculate that altered FlnA-PTPN12 interaction impairs PTPN12 targeting to the trailing tails of migrating cells and that locally impair p190RhoGAP tyrosine status, RhoA activity, and focal adhesion retraction.

Phosphorylated tyrosine (Y416) of Src, which participates in focal adhesion maturation and signaling, is another known target of PTPN12 [31]. We, thus, analyzed Src phosphorylation status in mutant FlnA-expressing cells. As shown on Figure 4C, phosphorylated Src-Y146 levels detected in FlnA-G288R and P637Q cells using pSrc-Y416 antibody almost doubled compared to FlnA-WT expressing cells (*p* < 0.01, *n* = 5).

## 4. Discussion

The tyrosine phosphatase PTPN12/PTP-PEST was previously shown to interact with FlnA through one of its proline-rich domains but the counterpart binding domain on FlnA remained undefined [28]. Here, we have uncovered that the first eight repeats of FlnA constitute a binding site for PTPN12/PTP-PEST and showed that the FlnA mutations associated to mitral valve prolapse that specifically target this region disrupt PTPN12/FlnA interaction.

PTPN12 is a ubiquitously-expressed phosphatase which has been implicated in immune response and vascular development in part because of its roles in cell-ECM interaction, cell migration, and small GTPases signaling pathways. Considering the large crossover of these pathways with those in which FlnA also participates, it is inciting to speculate that the disruption of their interaction could have a role in the development of MVP in FlnA mutation carriers. The fact that the three mutations tested here all knock down FlnA/PTPN12 interaction suggests, in addition, that similar molecular and cellular mechanisms are at work in the pathology. This is also consistent with the strong homogeneity of the physio-pathological traits observed in the patients, including the poly-valvular damages, the echographic features of the mitral valve prolapse, the early onset of the disease, and the absence of any other symptom. Additionally, not surprising and consistent with the shared, but specific, phenotype, all the mutations target the same narrow region (Igl-1–8 repeats) localized between the two actin binding domains of FlnA [15].

Previous studies identified Ras GTPase, SyK tyrosine kinase, PKC epsilon, and vimentin as binding partners of FlnA N-terminal Igl domains [18,19,32]. However, we did not detect these binding partners in our experiments (not shown). On the other hand, we showed that FlnA-WT does interact with PTPN12, whereas FlnA-G288R, P637Q, and H743P mutations knocked down the PTPN12/FlnA interactions in our two-hybrid assay. In addition, we confirmed this loss of interaction for FlnA-P637Q in both GST pulldown assay and co-immunoprecipitation experiments. The detailed molecular mechanisms of FlnA/PTPN12 interaction remain to be unveiled, but yeast two-hybrid assays strongly suggest a direct interaction between the two partners. Obviously, because of the large crossover in the signaling pathways in which both FlnA and PTPN12 participate they also probably interact through long-range indirect interactions, in large signaling protein complexes. As far as “direct” interaction is concerned, it is tempting to speculate that it specifically involves the N-terminal region of FlnA. Indeed, on one hand, sequence-based classification of FlnA repeats indicated the Igl-4 (targeted by P637Q), and Igl-1 and 5 (targeted by G288R, V711D, and H743P mutations, respectively) belong to distinct repeat subgroups containing other repeats (e.g., subclass C contains Igl repeats one, two, three, and five, while subclass A contains Igl-4 in addition to Igl-9, 12, 17, 19, 21, 23) suggesting PTPN12 may bind other FlnA domains [33]. On the other hand, recent studies showed Igl-3–5 repeats constitute a peculiar three-domain interaction module whose structure is most probably impeded by the mutations. Glycine-288 (G288) is located in Igl-1 at the end of the first β-strand (the A strand) and is the first residue of a GPG motif that makes a helical turn in most Filamin Igl domains (e.g., Igl-16, 18, and 21). Interestingly, in three Filamin Igl domains where this motif is missing, the A-strand is not folded with the rest of the domain [34]. Thus, it is possible that the G288R mutation changes the conformation of Igl-1. Proline-637 (P637) and V711 are residues that point towards the hydrophobic core of Igl-4 and Igl-5, respectively. Substitution of these aliphatic residues with charged side chains in the P637Q and V711D mutations may change the stability of individual domains and also affect the function of the closely-interacting Igl-3–5 triplet [35,36]. Together, these clues suggest FlnA Igl-1–8 constitute a peculiar interaction domain on Filamin A and the major binding site of PTPN12. Future studies will be necessary to ascertain this hypothesis and determine whether the mutations affect the structure and/or mechanosensitivity of this interaction module.

PTP-PEST is a cytosolic ubiquitous protein tyrosine phosphatase (PTP) that contains, in addition to its catalytic domain, several protein-protein interaction domains that allow it to interface with several signaling pathways. Among others, PTP-PEST was shown to be recruited to the EFG receptor signaling complex [20]. However, previous studies essentially pointed to PTPN12 as a key regulator of cellular motility and cytoskeleton dynamics involved in immunity [21], cancer [20], and development [37]. PTPN12 invalidation is embryologically lethal in mice due to important vascular defects and un-successful liver formation [37]. In fact, PTPN12 is not required for endothelial cell differentiation and proliferation, nor the regulation of endothelial permeability, but is required for integrin-mediated adhesion and migration of endothelial cells [38]. This is in agreement with previous studies showing that PTPN12 substrates include the focal adhesion associated proteins Src kinase, p130Cas, and paxillin, as well as small GTPase regulators [22,29,39]. Importantly, FlnA is known to also be intimately associated with focal adhesion signaling pathway regulation and, indeed, we showed previously that MVP-FlnA mutations deregulate Rho/Rac1 balance and affect cellular spreading and migration resulting from an increased RhoA activity [24]. This is consistent with the lower activity of p190RhoGAP we detected in FlnA-P637Q-expressing cells in the present study. On the other hand, we also noticed that migrating mutant FlnA-expressing cells exhibited long trailing tails typically observed in PTPN12-depleted cells where p190RhoGAP activity is increased [29]. To reconcile these apparent discrepancies, one can speculate that FlnA/PTPN12 interaction participate to the targeting of PTPN12 to specific membrane domains and. thus. to the polarized GTPase regulation in migrating cells. as was recently demonstrated for the PTPN12/β8 integrin complex [22]. Consistent with this idea, such targeting properties to specific membrane domains were already ascribed to FlnA for one of its binding partner; FilGAP [40].

Interestingly, and coherent with the remarkable restriction of the FlnA mutations’ associated defects to cardiac valves that are submitted to intense hemodynamic stresses, the mechanical stress may also modulate PTPN12/FlnA mutation. In fact, the local tension applied to the actin cytoskeleton was shown to modulate FlnA affinity for its binding partners FilGAP and β7 integrin [41,42]. Whether the mechanical stress also impacts the structure, the binding interface of Igl repeats (1–8) and FlnA/PTPN12 interactions remains to be elucidated [35,36].

## 5. Conclusions

In conclusion, we have identified here PTPN12 as a specific binding partner of the FlnA domain targeted by the MVP-associated FlnA mutations and showed the later impede FlnA/PTPN12 interaction. The key role of PTPN12 in the regulation of focal adhesion, ECM/cell crosstalk and cellular mechanical stress response signaling pathway make PTPN12 an interesting candidate involved in the physiological processes of FlnA-associated MVP.
